# Supplementary material for: Clasnip: a web-based intraspecies classifier and multi-locus sequence typing for pathogenic microorganisms using fragmented sequences
Source: PeerJ. 2023 Jan 9;11:e14490. doi: 10.7717/peerj.14490 (PMC9835710; doi:10.7717/peerj.14490)
Supplement: Supplemental Information 1 [file peerj-11-14490-s001.docx]

**Table S1:**

**The accession numbers of CLso samples used in Clasnip database building.**

| **Haplotype** | **Region** | **NCBI Accession ID** |
| --- | --- | --- |
| A | 16S, 16S-23S | EU834130.1 |
| A | 50S | EU834131.1 |
| A | Genome | JNVH01000001.1 |
| A | Genome | JQIG01000001.1 |
| A | Genome | JMTK01000001.1 |
| A | Genome | LLVZ01000001.1 |
| A | 16S-23S | JX624236.1 |
| A | 50S | JX624241.1 |
| A | 16S | JX624246.1 |
| A | 16S | KR935886.1 |
| A | 16S | KR935887.1 |
| A | 16S | MK726035.1 |
| A | 16S | MK726036.1 |
| B | Genome | CP002371.1 |
| B | 16S | JF811596.1 |
| B | 16S | JF811597.1 |
| B | 50S | JF811598.1 |
| B | 50S | JF811599.1 |
| B | 16S | KU588194.1 |
| B | 16S | KU588195.1 |
| B | 16S | MK726032.1 |
| B | 16S | MK726033.1 |
| B | 16S | MK726034.1 |
| B | 16S | MK726037.1 |
| C | Genome | LVWB01000001.1 |
| C | Genome | LWEB01000001.1 |
| C | 16S | KF170062.1 |
| C | 16S | KF170063.1 |
| C | 16S | KF170065.1 |
| C | 16S | KF170066.1 |
| C | 16S-23S | KJ584927.1 |
| C | 16S-23S | KJ584928.1 |
| C | 16S-23S | KJ584930.1 |
| C | 16S-23S | KJ584931.1 |
| C | 16S, 16S-23S, 5S | KX431889.1 |
| C | 16S, 16S-23S, 5S | KX431890.1 |
| C | 16S, 16S-23S, 5S | KX431891.1 |
| C | 16S-23S | KY619975.1 |
| C | 16S-23S | KY619976.1 |
| C | 16S-23S | KY619978.1 |
| C | 50S | KY619987.1 |
| C | 50S | KY619988.1 |
| C | 50S | KY619990.1 |
| C | 16S | KY624595.1 |
| C | 16S-23S | MF421724.1 |
| C | 16S-23S | MF421725.1 |
| C | 16S-23S | MF421726.1 |
| C | 16S-23S | MF421727.1 |
| C | 16S-23S | MF421728.1 |
| C | 16S-23S | MF421729.1 |
| C | 16S-23S | MF421730.1 |
| C | 16S | MG701017.1 |
| C | 16S-23S | MG701019.1 |
| C | 16S-23S | MG701020.1 |
| C | 50S | MG701024.1 |
| C | 50S | MG701030.1 |
| C | 50S | MG701031.1 |
| C | 50S | MG701032.1 |
| C | 50S | MG701033.1 |
| C | 50S | MG701034.1 |
| C | 50S | MG701035.1 |
| C | 50S | MG701036.1 |
| C | 50S | MG701037.1 |
| C | 50S | MG701038.1 |
| C | 50S | MG701039.1 |
| C | 50S | MG701040.1 |
| C | 50S | MG701041.1 |
| C | 50S | MG701042.1 |
| C | 50S | MG701043.1 |
| C | 50S | MG701044.1 |
| C | 50S | MG701045.1 |
| C | 50S | MG701046.1 |
| C | 50S | MG701047.1 |
| C | 50S | MG701048.1 |
| C | 50S | MG701049.1 |
| C | 50S | MG701050.1 |
| C | 50S | MG701051.1 |
| C | 50S | MG701052.1 |
| C | 50S | MG701053.1 |
| C | 50S | MG701054.1 |
| C | adk | MG704922.1 |
| C | adk | MG704924.1 |
| C | adk | MG704925.1 |
| C | adk | MG704926.1 |
| C | adk | MG704927.1 |
| C | adk | MG704928.1 |
| C | adk | MG704929.1 |
| C | adk | MG704930.1 |
| C | adk | MG704931.1 |
| C | adk | MG704932.1 |
| C | adk | MG704933.1 |
| C | adk | MG704937.1 |
| C | adk | MG704938.1 |
| C | adk | MG704939.1 |
| C | adk | MG704942.1 |
| C | adk | MG704943.1 |
| C | adk | MG704944.1 |
| C | adk | MG704945.1 |
| C | adk | MG704946.1 |
| C | adk | MG704947.1 |
| C | adk | MG704948.1 |
| C | adk | MG704949.1 |
| C | adk | MG704950.1 |
| C | adk | MG704951.1 |
| C | adk | MG704952.1 |
| C | adk | MG704953.1 |
| C | adk | MG704954.1 |
| C | adk | MG704955.1 |
| C | adk | MG704956.1 |
| C | adk | MG704957.1 |
| C | adk | MG704958.1 |
| C | atpA | MG704959.1 |
| C | atpA | MG704961.1 |
| C | atpA | MG704962.1 |
| C | atpA | MG704963.1 |
| C | atpA | MG704964.1 |
| C | atpA | MG704965.1 |
| C | atpA | MG704966.1 |
| C | atpA | MG704967.1 |
| C | atpA | MG704968.1 |
| C | atpA | MG704969.1 |
| C | atpA | MG704970.1 |
| C | atpA | MG704974.1 |
| C | atpA | MG704975.1 |
| C | atpA | MG704976.1 |
| C | atpA | MG704979.1 |
| C | atpA | MG704980.1 |
| C | atpA | MG704981.1 |
| C | atpA | MG704982.1 |
| C | atpA | MG704983.1 |
| C | atpA | MG704984.1 |
| C | atpA | MG704985.1 |
| C | atpA | MG704986.1 |
| C | atpA | MG704987.1 |
| C | atpA | MG704988.1 |
| C | atpA | MG704989.1 |
| C | atpA | MG704990.1 |
| C | atpA | MG704991.1 |
| C | atpA | MG704992.1 |
| C | atpA | MG704993.1 |
| C | atpA | MG704994.1 |
| C | atpA | MG704995.1 |
| C | fbpA | MG704996.1 |
| C | fbpA | MG704998.1 |
| C | fbpA | MG704999.1 |
| C | fbpA | MG705000.1 |
| C | fbpA | MG705001.1 |
| C | fbpA | MG705002.1 |
| C | fbpA | MG705003.1 |
| C | fbpA | MG705004.1 |
| C | fbpA | MG705005.1 |
| C | fbpA | MG705006.1 |
| C | fbpA | MG705007.1 |
| C | fbpA | MG705011.1 |
| C | fbpA | MG705012.1 |
| C | fbpA | MG705013.1 |
| C | fbpA | MG705016.1 |
| C | fbpA | MG705017.1 |
| C | fbpA | MG705018.1 |
| C | fbpA | MG705019.1 |
| C | fbpA | MG705020.1 |
| C | fbpA | MG705021.1 |
| C | fbpA | MG705022.1 |
| C | fbpA | MG705023.1 |
| C | fbpA | MG705024.1 |
| C | fbpA | MG705025.1 |
| C | fbpA | MG705026.1 |
| C | fbpA | MG705027.1 |
| C | fbpA | MG705028.1 |
| C | fbpA | MG705029.1 |
| C | fbpA | MG705030.1 |
| C | fbpA | MG705031.1 |
| C | fbpA | MG705032.1 |
| C | ftsZ | MG705033.1 |
| C | ftsZ | MG705035.1 |
| C | ftsZ | MG705036.1 |
| C | ftsZ | MG705037.1 |
| C | ftsZ | MG705038.1 |
| C | ftsZ | MG705039.1 |
| C | ftsZ | MG705040.1 |
| C | ftsZ | MG705041.1 |
| C | ftsZ | MG705042.1 |
| C | ftsZ | MG705043.1 |
| C | ftsZ | MG705044.1 |
| C | ftsZ | MG705048.1 |
| C | ftsZ | MG705049.1 |
| C | ftsZ | MG705050.1 |
| C | ftsZ | MG705053.1 |
| C | ftsZ | MG705054.1 |
| C | ftsZ | MG705055.1 |
| C | ftsZ | MG705056.1 |
| C | ftsZ | MG705057.1 |
| C | ftsZ | MG705058.1 |
| C | ftsZ | MG705059.1 |
| C | ftsZ | MG705060.1 |
| C | ftsZ | MG705061.1 |
| C | ftsZ | MG705063.1 |
| C | ftsZ | MG705064.1 |
| C | ftsZ | MG705065.1 |
| C | ftsZ | MG705066.1 |
| C | ftsZ | MG705067.1 |
| C | ftsZ | MG705068.1 |
| C | ftsZ | MG705069.1 |
| C | glyA | MG705070.1 |
| C | glyA | MG705072.1 |
| C | glyA | MG705073.1 |
| C | glyA | MG705074.1 |
| C | glyA | MG705075.1 |
| C | glyA | MG705076.1 |
| C | glyA | MG705077.1 |
| C | glyA | MG705078.1 |
| C | glyA | MG705079.1 |
| C | glyA | MG705080.1 |
| C | glyA | MG705081.1 |
| C | glyA | MG705085.1 |
| C | glyA | MG705086.1 |
| C | glyA | MG705087.1 |
| C | glyA | MG705090.1 |
| C | glyA | MG705091.1 |
| C | glyA | MG705092.1 |
| C | glyA | MG705093.1 |
| C | glyA | MG705094.1 |
| C | glyA | MG705095.1 |
| C | glyA | MG705096.1 |
| C | glyA | MG705097.1 |
| C | glyA | MG705098.1 |
| C | glyA | MG705099.1 |
| C | glyA | MG705100.1 |
| C | glyA | MG705101.1 |
| C | glyA | MG705102.1 |
| C | glyA | MG705103.1 |
| C | glyA | MG705104.1 |
| C | glyA | MG705105.1 |
| C | glyA | MG705106.1 |
| C | groEL | MG705107.1 |
| C | groEL | MG705109.1 |
| C | groEL | MG705110.1 |
| C | groEL | MG705111.1 |
| C | groEL | MG705112.1 |
| C | groEL | MG705113.1 |
| C | groEL | MG705114.1 |
| C | groEL | MG705115.1 |
| C | groEL | MG705116.1 |
| C | groEL | MG705117.1 |
| C | groEL | MG705118.1 |
| C | groEL | MG705122.1 |
| C | groEL | MG705123.1 |
| C | groEL | MG705124.1 |
| C | groEL | MG705127.1 |
| C | groEL | MG705128.1 |
| C | groEL | MG705129.1 |
| C | groEL | MG705130.1 |
| C | groEL | MG705131.1 |
| C | groEL | MG705132.1 |
| C | groEL | MG705133.1 |
| C | groEL | MG705134.1 |
| C | groEL | MG705135.1 |
| C | groEL | MG705136.1 |
| C | groEL | MG705137.1 |
| C | groEL | MG705138.1 |
| C | groEL | MG705139.1 |
| C | groEL | MG705140.1 |
| C | groEL | MG705141.1 |
| C | groEL | MG705142.1 |
| C | groEL | MG705143.1 |
| C | gyrB | MG705144.1 |
| C | gyrB | MG705146.1 |
| C | gyrB | MG705147.1 |
| C | gyrB | MG705148.1 |
| C | gyrB | MG705149.1 |
| C | gyrB | MG705150.1 |
| C | gyrB | MG705151.1 |
| C | gyrB | MG705152.1 |
| C | gyrB | MG705153.1 |
| C | gyrB | MG705154.1 |
| C | gyrB | MG705155.1 |
| C | gyrB | MG705159.1 |
| C | gyrB | MG705160.1 |
| C | gyrB | MG705161.1 |
| C | gyrB | MG705164.1 |
| C | gyrB | MG705165.1 |
| C | gyrB | MG705166.1 |
| C | gyrB | MG705167.1 |
| C | gyrB | MG705168.1 |
| C | gyrB | MG705169.1 |
| C | gyrB | MG705170.1 |
| C | gyrB | MG705171.1 |
| C | gyrB | MG705172.1 |
| C | gyrB | MG705173.1 |
| C | gyrB | MG705174.1 |
| C | gyrB | MG705175.1 |
| C | gyrB | MG705176.1 |
| C | gyrB | MG705177.1 |
| C | gyrB | MG705178.1 |
| C | gyrB | MG705179.1 |
| C | gyrB | MG705180.1 |
| Cras1a | 16S | MT229445.1 |
| Cras1a | 16S | MT229447.1 |
| Cras1a | 16S | MT229448.1 |
| Cras1a | 16S | MT229449.1 |
| Cras1a | 16S | MT229452.1 |
| Cras1a | 16S | MT229453.1 |
| Cras1a | 16S | MT229454.1 |
| Cras1a | 16S | MT229455.1 |
| Cras1a | 16S | MT229456.1 |
| Cras1a | 16S | MT229457.1 |
| Cras1a | 16S | MT229458.1 |
| Cras1a | 16S | MT229459.1 |
| Cras1a | 16S | MT229460.1 |
| Cras1a | 16S-23S | MT230487.1 |
| Cras1a | 16S-23S | MT230488.1 |
| Cras1a | 16S-23S | MT230489.1 |
| Cras1a | 16S-23S | MT230490.1 |
| Cras1a | 16S-23S | MT230492.1 |
| Cras1a | 16S-23S | MT230493.1 |
| Cras1a | 16S-23S | MT230494.1 |
| Cras1a | 16S-23S | MT230495.1 |
| Cras1a | 16S-23S | MT230498.1 |
| Cras1a | 16S-23S | MT230499.1 |
| Cras1a | 16S-23S | MT230500.1 |
| Cras1a | 16S-23S | MT230501.1 |
| Cras1a | 16S-23S | MT230502.1 |
| Cras1a | 16S-23S | MT230503.1 |
| Cras1a | 16S-23S | MT230504.1 |
| Cras1a | omp | MT238961.1 |
| Cras1a | omp | MT238962.1 |
| Cras1a | omp | MT238963.1 |
| Cras1a | omp | MT238964.1 |
| Cras1a | omp | MT238966.1 |
| Cras1a | omp | MT238967.1 |
| Cras1a | omp | MT238970.1 |
| Cras1a | omp | MT238971.1 |
| Cras1a | omp | MT238972.1 |
| Cras1a | omp | MT238973.1 |
| Cras1a | omp | MT238974.1 |
| Cras1a | 50S | MT249166.1 |
| Cras1a | 50S | MT249168.1 |
| Cras1a | 50S | MT249169.1 |
| Cras1a | 50S | MT249175.1 |
| Cras1a | 50S | MT249176.1 |
| Cras1a | 50S | MT249177.1 |
| Cras1a | 50S | MT249188.1 |
| Cras1a | 50S | MT249189.1 |
| Cras1a | 50S | MT249202.1 |
| Cras1a | 50S | MT249203.1 |
| Cras1a | 50S | MT249204.1 |
| Cras1a | 50S | MT249205.1 |
| Cras1a | 50S | MT249207.1 |
| Cras1a | 50S | MT249208.1 |
| Cras1a | 50S | MT249209.1 |
| Cras1a | 50S | MT249210.1 |
| Cras1a | 50S | MT249211.1 |
| Cras1a | 50S | MT249212.1 |
| Cras1b | 16S | MT229446.1 |
| Cras1b | 16S | MT229450.1 |
| Cras1b | 16S | MT229451.1 |
| Cras1b | 16S-23S | MT230491.1 |
| Cras1b | 16S-23S | MT230496.1 |
| Cras1b | 16S-23S | MT230497.1 |
| Cras1b | omp | MT238965.1 |
| Cras1b | omp | MT238968.1 |
| Cras1b | omp | MT238969.1 |
| Cras1b | 50S | MT249171.1 |
| Cras1b | 50S | MT249178.1 |
| Cras1b | 50S | MT249217.1 |
| Cras2 | 16S | MT229461.1 |
| Cras2 | 16S | MT229462.1 |
| Cras2 | 16S | MT229463.1 |
| Cras2 | 16S-23S | MT230506.1 |
| Cras2 | 16S-23S | MT230507.1 |
| Cras2 | 16S-23S | MT230508.1 |
| Cras2 | 16S-23S | MT230509.1 |
| Cras2 | omp | MT238975.1 |
| Cras2 | omp | MT238976.1 |
| Cras2 | omp | MT238977.1 |
| Cras2 | omp | MT238978.1 |
| Cras2 | 50S | MT249167.1 |
| Cras2 | 50S | MT249172.1 |
| Cras2 | 50S | MT249190.1 |
| Cras2 | 50S | MT249206.1 |
| D | Genome | PKRU02000001.1 |
| D | 16S | KX163276.1 |
| D | 16S | KX163277.1 |
| D | 50S | KX618639.1 |
| D | 50S | KX618640.1 |
| D | 16S-23S | KX752575.1 |
| D | 16S-23S | KX752580.1 |
| D | 50S | KX752582.1 |
| D | 16S | KX752587.1 |
| D | 16S-23S | KY486280.1 |
| D | 16S-23S | KY486281.1 |
| D | 16S-23S | KY486282.1 |
| D | 16S-23S | KY486283.1 |
| D | 16S-23S | KY486284.1 |
| D | 16S-23S | KY486285.1 |
| D | 16S-23S | KY486286.1 |
| D | 16S-23S | KY486287.1 |
| D | 16S-23S | KY486288.1 |
| D | 16S-23S | KY486289.1 |
| D | 16S-23S | KY486290.1 |
| D | 16S-23S | KY486291.1 |
| D | 16S-23S | KY486292.1 |
| D | 50S | KY486293.1 |
| D | 50S | KY486294.1 |
| D | 50S | KY486295.1 |
| D | 16S | KY486296.1 |
| D | 16S-23S | KY619977.1 |
| D | 16S-23S | KY619979.1 |
| D | 16S-23S | KY619981.1 |
| D | 16S-23S | KY619982.1 |
| D | 16S-23S | KY619983.1 |
| D | 50S | KY619985.1 |
| D | 50S | KY619989.1 |
| D | 16S | KY624596.1 |
| D | 50S | KY753132.1 |
| D | 50S | KY777462.1 |
| D | 50S | MG657027.1 |
| D | 16S | MG701014.1 |
| D | 50S | MG701021.1 |
| D | adk | MG704936.1 |
| D | atpA | MG704973.1 |
| D | fbpA | MG705010.1 |
| D | ftsZ | MG705047.1 |
| D | glyA | MG705084.1 |
| D | groEL | MG705121.1 |
| D | gyrB | MG705158.1 |
| D | 16S-23S | MG911708.1 |
| D | 16S-23S | MG911709.1 |
| D | 16S | MG911711.1 |
| D | 16S | MG911712.1 |
| D | 50S | MG911714.1 |
| D | 50S | MG911715.1 |
| D | 16S | MH061376.1 |
| D | 16S-23S | MH061377.1 |
| D | 50S | MH061378.1 |
| D | 50S | MH748578.1 |
| E | 16S-23S | KX752576.1 |
| E | 16S-23S | KX752577.1 |
| E | 16S-23S | KX752578.1 |
| E | 16S-23S | KX752579.1 |
| E | 16S-23S | KX752581.1 |
| E | 50S | KX752583.1 |
| E | 50S | KX752584.1 |
| E | 50S | KX752585.1 |
| E | 50S | KX752586.1 |
| E | 16S | KX752588.1 |
| E | 16S | KX752589.1 |
| E | 16S-23S | KY619980.1 |
| E | 50S | KY619984.1 |
| E | 50S | KY619986.1 |
| E | 16S | KY619991.1 |
| E | 16S | KY619992.1 |
| E | 50S | KY777461.1 |
| E | 16S-23S | MG911710.1 |
| E | 16S | MG911713.1 |
| E | 50S | MG911716.1 |
| F | 16S | MH259699.1 |
| F | 50S | MH259700.1 |
| F | omp | MH259701.1 |
| G | 16S | MN256493.1 |
| G | 16S | MN256494.1 |
| G | 16S | MN256495.1 |
| G | 16S-23S | MN256496.1 |
| G | 16S-23S | MN256497.1 |
| G | 16S-23S | MN256498.1 |
| G | 50S | MN256499.1 |
| G | 50S | MN256500.1 |
| G | 50S | MN256501.1 |
| G | 50S | MN256502.1 |
| G | adk | MN256503.1 |
| G | adk | MN256504.1 |
| G | adk | MN256505.1 |
| G | adk | MN256506.1 |
| G | atpA | MN256507.1 |
| G | atpA | MN256508.1 |
| G | atpA | MN256509.1 |
| G | atpA | MN256510.1 |
| G | fbpA | MN256511.1 |
| G | fbpA | MN256512.1 |
| G | fbpA | MN256513.1 |
| G | fbpA | MN256514.1 |
| G | ftsZ | MN256515.1 |
| G | ftsZ | MN256516.1 |
| G | ftsZ | MN256517.1 |
| G | ftsZ | MN256518.1 |
| G | glyA | MN256519.1 |
| G | glyA | MN256520.1 |
| G | glyA | MN256521.1 |
| G | glyA | MN256522.1 |
| G | groEL | MN256523.1 |
| G | groEL | MN256524.1 |
| G | groEL | MN256525.1 |
| G | groEL | MN256526.1 |
| G | gyrB | MN256527.1 |
| G | gyrB | MN256528.1 |
| G | gyrB | MN256529.1 |
| G | gyrB | MN256530.1 |
| H-Con | 16S | KT354971.1 |
| H-Con | 16S | KT354975.1 |
| H | adk | MK800158.1 |
| H | atpA | MK800159.1 |
| H | fbpA | MK800160.1 |
| H | ftsZ | MK800161.1 |
| H | ftsZ | MK800162.1 |
| H | glyA | MK800163.1 |
| H | groEL | MK800164.1 |
| H | gyrB | MK800165.1 |
| H | gyrB | MK800166.1 |
| H | 16S | MK800167.1 |
| H | 16S-23S | MK800168.1 |
| H | 50S | MK800169.1 |
| U | 16S | MG701016.1 |
| U | 16S-23S | MG701018.1 |
| U | 50S | MG701023.1 |
| U | 50S | MG701026.1 |
| U | 50S | MG701027.1 |
| U | 50S | MG701028.1 |
| U | 50S | MG701029.1 |
| U | adk | MG704923.2 |
| U | adk | MG704934.2 |
| U | adk | MG704935.2 |
| U | adk | MG704940.2 |
| U | adk | MG704941.2 |
| U | atpA | MG704960.1 |
| U | atpA | MG704971.1 |
| U | atpA | MG704972.1 |
| U | atpA | MG704977.1 |
| U | atpA | MG704978.1 |
| U | fbpA | MG704997.1 |
| U | fbpA | MG705008.1 |
| U | fbpA | MG705009.1 |
| U | fbpA | MG705014.1 |
| U | fbpA | MG705015.1 |
| U | ftsZ | MG705034.1 |
| U | ftsZ | MG705045.1 |
| U | ftsZ | MG705046.1 |
| U | ftsZ | MG705051.1 |
| U | ftsZ | MG705052.1 |
| U | glyA | MG705071.1 |
| U | glyA | MG705082.1 |
| U | glyA | MG705083.1 |
| U | glyA | MG705088.1 |
| U | glyA | MG705089.1 |
| U | groEL | MG705108.1 |
| U | groEL | MG705119.1 |
| U | groEL | MG705120.1 |
| U | groEL | MG705125.1 |
| U | groEL | MG705126.1 |
| U | gyrB | MG705145.1 |
| U | gyrB | MG705156.1 |
| U | gyrB | MG705157.1 |
| U | gyrB | MG705162.1 |
| U | gyrB | MG705163.1 |
