# Supplementary material for: Clasnip: a web-based intraspecies classifier and multi-locus sequence typing for pathogenic microorganisms using fragmented sequences
Source: PeerJ. 2023 Jan 9;11:e14490. doi: 10.7717/peerj.14490 (PMC9835710; doi:10.7717/peerj.14490)
Supplement: Supplemental Information 2 — TPR = Sensitivity, Recall, Hit Rate, True Positive Rate. TNR = Specificity, Selectivity, True Negative Rate. PPV = Precision, Positive Predictive Value. NPV = Negative Predictive Value. FNR = Miss Rate, False Negative Rate. FPR = Fall-out, False Positive Rate. FDR = False Discovery Rate. FOR = False Omission Rate. ACC = Accuracy. F1 = the harmonic mean of precision and sensitivity. [file peerj-11-14490-s002.docx]

**Table S2:**

**Performance statistics of the CLso and PVY databases.**

TPR = Sensitivity, Recall, Hit Rate, True Positive Rate.

TNR = Specificity, Selectivity, True Negative Rate.

PPV = Precision, Positive Predictive Value.

NPV = Negative Predictive Value.

FNR = Miss Rate, False Negative Rate.

FPR = Fall-out, False Positive Rate.

FDR = False Discovery Rate.

FOR = False Omission Rate.

ACC = Accuracy.

F1 = the harmonic mean of precision and sensitivity.

| **Database** | **Group** | **TPR** | **TNR** | **PPV** | **NPV** | **FNR** | **FPR** | **FDR** | **FOR** | **ACC** | **F1** |
| --- | --- | --- | --- | --- | --- | --- | --- | --- | --- | --- | --- |
| CLso 16S | A | 1 | 0.998 | 0.929 | 1 | 0 | 0.002 | 0.071 | 0 | 0.998 | 0.963 |
|  | B | 0.909 | 1 | 1 | 0.998 | 0.091 | 0 | 0 | 0.002 | 0.998 | 0.952 |
|  | C | 0.996 | 1 | 1 | 0.996 | 0.004 | 0 | 0 | 0.004 | 0.998 | 0.998 |
|  | Cras1a | 1 | 1 | 1 | 1 | 0 | 0 | 0 | 0 | 1 | 1 |
|  | Cras1b | 1 | 1 | 1 | 1 | 0 | 0 | 0 | 0 | 1 | 1 |
|  | Cras2 | 1 | 1 | 1 | 1 | 0 | 0 | 0 | 0 | 1 | 1 |
|  | D | 1 | 1 | 1 | 1 | 0 | 0 | 0 | 0 | 1 | 1 |
|  | E | 1 | 1 | 1 | 1 | 0 | 0 | 0 | 0 | 1 | 1 |
|  | F | 1 | 1 | 1 | 1 | 0 | 0 | 0 | 0 | 1 | 1 |
|  | G | 1 | 1 | 1 | 1 | 0 | 0 | 0 | 0 | 1 | 1 |
|  | H | 1 | 1 | 1 | 1 | 0 | 0 | 0 | 0 | 1 | 1 |
|  | H-Con | 1 | 1 | 1 | 1 | 0 | 0 | 0 | 0 | 1 | 1 |
|  | U | 1 | 0.998 | 0.977 | 1 | 0 | 0.002 | 0.023 | 0 | 0.998 | 0.988 |
| CLso 16-23S | A | 1 | 1 | 1 | 1 | 0 | 0 | 0 | 0 | 1 | 1 |
|  | B | 1 | 1 | 1 | 1 | 0 | 0 | 0 | 0 | 1 | 1 |
|  | C | 1 | 1 | 1 | 1 | 0 | 0 | 0 | 0 | 1 | 1 |
|  | Cras1a | 0.982 | 1 | 1 | 0.998 | 0.018 | 0 | 0 | 0.002 | 0.998 | 0.991 |
|  | Cras1b | 1 | 0.998 | 0.923 | 1 | 0 | 0.002 | 0.077 | 0 | 0.998 | 0.96 |
|  | Cras2 | 1 | 1 | 1 | 1 | 0 | 0 | 0 | 0 | 1 | 1 |
|  | D | 1 | 1 | 1 | 1 | 0 | 0 | 0 | 0 | 1 | 1 |
|  | E | 1 | 1 | 1 | 1 | 0 | 0 | 0 | 0 | 1 | 1 |
|  | F | 1 | 1 | 1 | 1 | 0 | 0 | 0 | 0 | 1 | 1 |
|  | G | 1 | 1 | 1 | 1 | 0 | 0 | 0 | 0 | 1 | 1 |
|  | H | 1 | 1 | 1 | 1 | 0 | 0 | 0 | 0 | 1 | 1 |
|  | H-Con | 1 | 1 | 1 | 1 | 0 | 0 | 0 | 0 | 1 | 1 |
|  | U | 1 | 1 | 1 | 1 | 0 | 0 | 0 | 0 | 1 | 1 |
| CLso 50S | A | 1 | 1 | 1 | 1 | 0 | 0 | 0 | 0 | 1 | 1 |
|  | B | 1 | 1 | 1 | 1 | 0 | 0 | 0 | 0 | 1 | 1 |
|  | C | 1 | 1 | 1 | 1 | 0 | 0 | 0 | 0 | 1 | 1 |
|  | Cras1a | 1 | 1 | 1 | 1 | 0 | 0 | 0 | 0 | 1 | 1 |
|  | Cras1b | 1 | 1 | 1 | 1 | 0 | 0 | 0 | 0 | 1 | 1 |
|  | Cras2 | 1 | 1 | 1 | 1 | 0 | 0 | 0 | 0 | 1 | 1 |
|  | D | 1 | 1 | 1 | 1 | 0 | 0 | 0 | 0 | 1 | 1 |
|  | E | 1 | 1 | 1 | 1 | 0 | 0 | 0 | 0 | 1 | 1 |
|  | F | 1 | 1 | 1 | 1 | 0 | 0 | 0 | 0 | 1 | 1 |
|  | G | 1 | 1 | 1 | 1 | 0 | 0 | 0 | 0 | 1 | 1 |
|  | H | 1 | 1 | 1 | 1 | 0 | 0 | 0 | 0 | 1 | 1 |
|  | H-Con | 1 | 1 | 1 | 1 | 0 | 0 | 0 | 0 | 1 | 1 |
|  | U | 1 | 1 | 1 | 1 | 0 | 0 | 0 | 0 | 1 | 1 |
| CLso Genomic | A | 1 | 1 | 1 | 1 | 0 | 0 | 0 | 0 | 1 | 1 |
|  | B | 1 | 1 | 1 | 1 | 0 | 0 | 0 | 0 | 1 | 1 |
|  | C | 0.982 | 1 | 1 | 0.983 | 0.018 | 0 | 0 | 0.017 | 0.991 | 0.991 |
|  | Cras1a | 1 | 1 | 1 | 1 | 0 | 0 | 0 | 0 | 1 | 1 |
|  | Cras1b | 1 | 1 | 1 | 1 | 0 | 0 | 0 | 0 | 1 | 1 |
|  | Cras2 | 1 | 1 | 1 | 1 | 0 | 0 | 0 | 0 | 1 | 1 |
|  | D | 1 | 1 | 1 | 1 | 0 | 0 | 0 | 0 | 1 | 1 |
|  | E | 1 | 1 | 1 | 1 | 0 | 0 | 0 | 0 | 1 | 1 |
|  | F | 1 | 1 | 1 | 1 | 0 | 0 | 0 | 0 | 1 | 1 |
|  | G | 1 | 1 | 1 | 1 | 0 | 0 | 0 | 0 | 1 | 1 |
|  | H | 1 | 1 | 1 | 1 | 0 | 0 | 0 | 0 | 1 | 1 |
|  | H-Con | 1 | 1 | 1 | 1 | 0 | 0 | 0 | 0 | 1 | 1 |
|  | U | 1 | 0.992 | 0.913 | 1 | 0 | 0.008 | 0.087 | 0 | 0.993 | 0.955 |
| PVY Genomic | C | 1 | 1 | 1 | 1 | 0 | 0 | 0 | 0 | 1 | 1 |
|  | Chile3 | 1 | 1 | 1 | 1 | 0 | 0 | 0 | 0 | 1 | 1 |
|  | N | 1 | 1 | 1 | 1 | 0 | 0 | 0 | 0 | 1 | 1 |
|  | NTN | 1 | 1 | 1 | 1 | 0 | 0 | 0 | 0 | 1 | 1 |
|  | N:O | 1 | 1 | 1 | 1 | 0 | 0 | 0 | 0 | 1 | 1 |
|  | O | 1 | 1 | 1 | 1 | 0 | 0 | 0 | 0 | 1 | 1 |
|  | Poha | 1 | 1 | 1 | 1 | 0 | 0 | 0 | 0 | 1 | 1 |
