# Supplementary material for: Clasnip: a web-based intraspecies classifier and multi-locus sequence typing for pathogenic microorganisms using fragmented sequences
Source: PeerJ. 2023 Jan 9;11:e14490. doi: 10.7717/peerj.14490 (PMC9835710; doi:10.7717/peerj.14490)
Supplement: Supplemental Information 3 — TP (True Positive): a test result that correctly indicates the presence of a condition or characteristic. TN (True Negative): a test result that correctly indicates the absence of a condition or characteristic. FP (False Positive): a test result which wrongly indicates that a particular condition or attribute is present. FN (False Negative): a test result which wrongly indicates that a particular condition or attribute is absent. TPR = Sensitivity, Recall, Hit Rate, True Positive Rate. TNR = Specificity, Selectivity, True Negative Rate. PPV = Precision, Positive Predictive Value. NPV = Negative Predictive Value. FNR = Miss Rate, False Negative Rate. FPR = Fall-out, False Positive Rate. FDR = False Discovery Rate. FOR = False Omission Rate. ACC = Accuracy. F1 = the harmonic mean of precision and sensitivity. [file peerj-11-14490-s003.docx]

**Table S3:**

**Cross-validation performance statistics of the CLso and PVY databases.**

TP (True Positive): a test result that correctly indicates the presence of a condition or characteristic.

TN (True Negative): a test result that correctly indicates the absence of a condition or characteristic.

FP (False Positive): a test result which wrongly indicates that a particular condition or attribute is present.

FN (False Negative): a test result which wrongly indicates that a particular condition or attribute is absent.

TPR = Sensitivity, Recall, Hit Rate, True Positive Rate.

TNR = Specificity, Selectivity, True Negative Rate.

PPV = Precision, Positive Predictive Value.

NPV = Negative Predictive Value.

FNR = Miss Rate, False Negative Rate.

FPR = Fall-out, False Positive Rate.

FDR = False Discovery Rate.

FOR = False Omission Rate.

ACC = Accuracy.

F1 = the harmonic mean of precision and sensitivity.

| **Database** | **Cross Validation** | **Group** | **TP** | **FP** | **TN** | **FN** | **TPR** | **TNR** | **PPV** | **NPV** | **FNR** | **FPR** | **FDR** | **FOR** | **ACC** | **F1** |
| --- | --- | --- | --- | --- | --- | --- | --- | --- | --- | --- | --- | --- | --- | --- | --- | --- |
| CLso 16S | Training | A | 5 ± 0 | 0.17 ± 0.41 | 29.3 ± 2.0 | 0 ± 0 | 1 ± 0 | 0.994 ± 0.015 | 0.972 ± 0.068 | 1 ± 0 | 0 ± 0 | 0.006 ± 0.015 | 0.028 ± 0.068 | 0 ± 0 | 0.995 ± 0.013 | 0.985 ± 0.037 |
|  |  | B | 4.5 ± 0.55 | 0 ± 0 | 30.0 ± 1.5 | 0 ± 0 | 1 ± 0 | 1 ± 0 | 1 ± 0 | 1 ± 0 | 0 ± 0 | 0 ± 0 | 0 ± 0 | 0 ± 0 | 1 ± 0 | 1 ± 0 |
|  |  | C | 5.33 ± 0.82 | 0 ± 0 | 29.0 ± 1.5 | 0.17 ± 0.41 | 0.967 ± 0.082 | 1 ± 0 | 1 ± 0 | 0.994 ± 0.015 | 0.033 ± 0.082 | 0 ± 0 | 0 ± 0 | 0.006 ± 0.015 | 0.995 ± 0.013 | 0.981 ± 0.045 |
|  |  | Cras1a | 6.5 ± 0.55 | 0 ± 0 | 28.0 ± 1.4 | 0 ± 0 | 1 ± 0 | 1 ± 0 | 1 ± 0 | 1 ± 0 | 0 ± 0 | 0 ± 0 | 0 ± 0 | 0 ± 0 | 1 ± 0 | 1 ± 0 |
|  |  | Cras1b | 1.5 ± 0.55 | 0 ± 0 | 33.0 ± 1.3 | 0 ± 0 | 1 ± 0 | 1 ± 0 | 1 ± 0 | 1 ± 0 | 0 ± 0 | 0 ± 0 | 0 ± 0 | 0 ± 0 | 1 ± 0 | 1 ± 0 |
|  |  | Cras2 | 1.5 ± 0.55 | 0 ± 0 | 33.0 ± 1.4 | 0 ± 0 | 1 ± 0 | 1 ± 0 | 1 ± 0 | 1 ± 0 | 0 ± 0 | 0 ± 0 | 0 ± 0 | 0 ± 0 | 1 ± 0 | 1 ± 0 |
|  |  | D | 5 ± 0 | 0 ± 0 | 29.5 ± 1.6 | 0 ± 0 | 1 ± 0 | 1 ± 0 | 1 ± 0 | 1 ± 0 | 0 ± 0 | 0 ± 0 | 0 ± 0 | 0 ± 0 | 1 ± 0 | 1 ± 0 |
|  |  | E | 2.5 ± 0.55 | 0 ± 0 | 32.0 ± 1.9 | 0 ± 0 | 1 ± 0 | 1 ± 0 | 1 ± 0 | 1 ± 0 | 0 ± 0 | 0 ± 0 | 0 ± 0 | 0 ± 0 | 1 ± 0 | 1 ± 0 |
|  |  | G | 1.5 ± 0.55 | 0 ± 0 | 33.0 ± 1.3 | 0 ± 0 | 1 ± 0 | 1 ± 0 | 1 ± 0 | 1 ± 0 | 0 ± 0 | 0 ± 0 | 0 ± 0 | 0 ± 0 | 1 ± 0 | 1 ± 0 |
|  |  | H-Con | 1 ± 0 | 0 ± 0 | 33.5 ± 1.6 | 0 ± 0 | 1 ± 0 | 1 ± 0 | 1 ± 0 | 1 ± 0 | 0 ± 0 | 0 ± 0 | 0 ± 0 | 0 ± 0 | 1 ± 0 | 1 ± 0 |
|  | Test | A | 4.67 ± 0.52 | 0.83 ± 0.75 | 28.7 ± 1.5 | 0.33 ± 0.52 | 0.93 ± 0.1 | 0.972 ± 0.024 | 0.86 ± 0.12 | 0.989 ± 0.018 | 0.07 ± 0.1 | 0.028 ± 0.024 | 0.14 ± 0.12 | 0.011 ± 0.018 | 0.966 ± 0.028 | 0.89 ± 0.094 |
|  |  | B | 4.0 ± 0.89 | 0 ± 0 | 30.0 ± 1.5 | 0.5 ± 0.55 | 0.88 ± 0.13 | 1 ± 0 | 1 ± 0 | 0.983 ± 0.018 | 0.12 ± 0.13 | 0 ± 0 | 0 ± 0 | 0.017 ± 0.018 | 0.985 ± 0.016 | 0.934 ± 0.073 |
|  |  | C | 5.17 ± 0.75 | 0.67 ± 0.52 | 28.3 ± 1.8 | 0.33 ± 0.82 | 0.94 ± 0.14 | 0.977 ± 0.018 | 0.893 ± 0.084 | 0.99 ± 0.025 | 0.06 ± 0.14 | 0.023 ± 0.018 | 0.107 ± 0.084 | 0.01 ± 0.025 | 0.971 ± 0.017 | 0.908 ± 0.064 |
|  |  | Cras1a | 6.5 ± 0.55 | 0 ± 0 | 28.0 ± 1.4 | 0 ± 0 | 1 ± 0 | 1 ± 0 | 1 ± 0 | 1 ± 0 | 0 ± 0 | 0 ± 0 | 0 ± 0 | 0 ± 0 | 1 ± 0 | 1 ± 0 |
|  |  | Cras1b | 1.5 ± 0.55 | 0 ± 0 | 33.0 ± 1.3 | 0 ± 0 | 1 ± 0 | 1 ± 0 | 1 ± 0 | 1 ± 0 | 0 ± 0 | 0 ± 0 | 0 ± 0 | 0 ± 0 | 1 ± 0 | 1 ± 0 |
|  |  | Cras2 | 1.5 ± 0.55 | 0 ± 0 | 33.0 ± 1.4 | 0 ± 0 | 1 ± 0 | 1 ± 0 | 1 ± 0 | 1 ± 0 | 0 ± 0 | 0 ± 0 | 0 ± 0 | 0 ± 0 | 1 ± 0 | 1 ± 0 |
|  |  | D | 4.67 ± 0.52 | 0 ± 0 | 29.5 ± 1.6 | 0.33 ± 0.52 | 0.93 ± 0.1 | 1 ± 0 | 1 ± 0 | 0.989 ± 0.018 | 0.07 ± 0.1 | 0 ± 0 | 0 ± 0 | 0.011 ± 0.018 | 0.99 ± 0.015 | 0.963 ± 0.057 |
|  |  | E | 2.5 ± 0.55 | 0 ± 0 | 32.0 ± 1.9 | 0 ± 0 | 1 ± 0 | 1 ± 0 | 1 ± 0 | 1 ± 0 | 0 ± 0 | 0 ± 0 | 0 ± 0 | 0 ± 0 | 1 ± 0 | 1 ± 0 |
|  |  | G | 1.5 ± 0.55 | 0 ± 0 | 33.0 ± 1.3 | 0 ± 0 | 1 ± 0 | 1 ± 0 | 1 ± 0 | 1 ± 0 | 0 ± 0 | 0 ± 0 | 0 ± 0 | 0 ± 0 | 1 ± 0 | 1 ± 0 |
|  |  | H-Con | 1 ± 0 | 0 ± 0 | 33.5 ± 1.6 | 0 ± 0 | 1 ± 0 | 1 ± 0 | 1 ± 0 | 1 ± 0 | 0 ± 0 | 0 ± 0 | 0 ± 0 | 0 ± 0 | 1 ± 0 | 1 ± 0 |
| CLso 16-23S | Training | A | 3 ± 0 | 0 ± 0 | 38.5 ± 1.6 | 0 ± 0 | 1 ± 0 | 1 ± 0 | 1 ± 0 | 1 ± 0 | 0 ± 0 | 0 ± 0 | 0 ± 0 | 0 ± 0 | 1 ± 0 | 1 ± 0 |
|  |  | C | 10.5 ± 0.55 | 0 ± 0 | 31.0 ± 1.3 | 0 ± 0 | 1 ± 0 | 1 ± 0 | 1 ± 0 | 1 ± 0 | 0 ± 0 | 0 ± 0 | 0 ± 0 | 0 ± 0 | 1 ± 0 | 1 ± 0 |
|  |  | Cras1a | 6.7 ± 1.0 | 0 ± 0 | 34.0 ± 1.4 | 0.8 ± 1.2 | 0.89 ± 0.15 | 1 ± 0 | 1 ± 0 | 0.976 ± 0.033 | 0.11 ± 0.15 | 0 ± 0 | 0 ± 0 | 0.024 ± 0.033 | 0.98 ± 0.029 | 0.938 ± 0.09 |
|  |  | Cras1b | 1.5 ± 0.55 | 0.8 ± 1.2 | 39.2 ± 1.9 | 0 ± 0 | 1 ± 0 | 0.979 ± 0.029 | 0.74 ± 0.32 | 1 ± 0 | 0 ± 0 | 0.021 ± 0.029 | 0.26 ± 0.32 | 0 ± 0 | 0.98 ± 0.029 | 0.81 ± 0.24 |
|  |  | Cras2 | 2 ± 0 | 0 ± 0 | 39.5 ± 1.6 | 0 ± 0 | 1 ± 0 | 1 ± 0 | 1 ± 0 | 1 ± 0 | 0 ± 0 | 0 ± 0 | 0 ± 0 | 0 ± 0 | 1 ± 0 | 1 ± 0 |
|  |  | D | 12 ± 0 | 0 ± 0 | 29.5 ± 1.6 | 0 ± 0 | 1 ± 0 | 1 ± 0 | 1 ± 0 | 1 ± 0 | 0 ± 0 | 0 ± 0 | 0 ± 0 | 0 ± 0 | 1 ± 0 | 1 ± 0 |
|  |  | E | 3.5 ± 0.55 | 0 ± 0 | 38.0 ± 1.5 | 0 ± 0 | 1 ± 0 | 1 ± 0 | 1 ± 0 | 1 ± 0 | 0 ± 0 | 0 ± 0 | 0 ± 0 | 0 ± 0 | 1 ± 0 | 1 ± 0 |
|  |  | G | 1.5 ± 0.55 | 0 ± 0 | 40.0 ± 1.4 | 0 ± 0 | 1 ± 0 | 1 ± 0 | 1 ± 0 | 1 ± 0 | 0 ± 0 | 0 ± 0 | 0 ± 0 | 0 ± 0 | 1 ± 0 | 1 ± 0 |
|  | Test | A | 2.83 ± 0.41 | 0 ± 0 | 38.5 ± 1.6 | 0.17 ± 0.41 | 0.94 ± 0.14 | 1 ± 0 | 1 ± 0 | 0.996 ± 0.01 | 0.06 ± 0.14 | 0 ± 0 | 0 ± 0 | 0.004 ± 0.01 | 0.996 ± 0.0097 | 0.967 ± 0.082 |
|  |  | C | 10.5 ± 0.55 | 0 ± 0 | 31.0 ± 1.3 | 0 ± 0 | 1 ± 0 | 1 ± 0 | 1 ± 0 | 1 ± 0 | 0 ± 0 | 0 ± 0 | 0 ± 0 | 0 ± 0 | 1 ± 0 | 1 ± 0 |
|  |  | Cras1a | 7.0 ± 0.89 | 0 ± 0 | 34.0 ± 1.4 | 0.5 ± 0.55 | 0.932 ± 0.075 | 1 ± 0 | 1 ± 0 | 0.986 ± 0.016 | 0.068 ± 0.075 | 0 ± 0 | 0 ± 0 | 0.014 ± 0.016 | 0.988 ± 0.013 | 0.963 ± 0.04 |
|  |  | Cras1b | 1.5 ± 0.55 | 0.5 ± 0.55 | 39.5 ± 1.6 | 0 ± 0 | 1 ± 0 | 0.987 ± 0.014 | 0.81 ± 0.22 | 1 ± 0 | 0 ± 0 | 0.013 ± 0.014 | 0.19 ± 0.22 | 0 ± 0 | 0.988 ± 0.013 | 0.88 ± 0.14 |
|  |  | Cras2 | 2 ± 0 | 0.17 ± 0.41 | 39.3 ± 1.6 | 0 ± 0 | 1 ± 0 | 0.996 ± 0.01 | 0.94 ± 0.14 | 1 ± 0 | 0 ± 0 | 0.004 ± 0.01 | 0.06 ± 0.14 | 0 ± 0 | 0.996 ± 0.0097 | 0.967 ± 0.082 |
|  |  | D | 12 ± 0 | 0 ± 0 | 29.5 ± 1.6 | 0 ± 0 | 1 ± 0 | 1 ± 0 | 1 ± 0 | 1 ± 0 | 0 ± 0 | 0 ± 0 | 0 ± 0 | 0 ± 0 | 1 ± 0 | 1 ± 0 |
|  |  | E | 3.5 ± 0.55 | 0 ± 0 | 38.0 ± 1.5 | 0 ± 0 | 1 ± 0 | 1 ± 0 | 1 ± 0 | 1 ± 0 | 0 ± 0 | 0 ± 0 | 0 ± 0 | 0 ± 0 | 1 ± 0 | 1 ± 0 |
|  |  | G | 1.5 ± 0.55 | 0 ± 0 | 40.0 ± 1.4 | 0 ± 0 | 1 ± 0 | 1 ± 0 | 1 ± 0 | 1 ± 0 | 0 ± 0 | 0 ± 0 | 0 ± 0 | 0 ± 0 | 1 ± 0 | 1 ± 0 |
| CLso 50S | Training | A | 3 ± 0 | 0 ± 0 | 46.5 ± 1.0 | 0 ± 0 | 1 ± 0 | 1 ± 0 | 1 ± 0 | 1 ± 0 | 0 ± 0 | 0 ± 0 | 0 ± 0 | 0 ± 0 | 1 ± 0 | 1 ± 0 |
|  |  | B | 1.5 ± 0.55 | 0 ± 0 | 48.0 ± 0.89 | 0 ± 0 | 1 ± 0 | 1 ± 0 | 1 ± 0 | 1 ± 0 | 0 ± 0 | 0 ± 0 | 0 ± 0 | 0 ± 0 | 1 ± 0 | 1 ± 0 |
|  |  | C | 15.5 ± 0.55 | 0 ± 0 | 34.0 ± 0.89 | 0 ± 0 | 1 ± 0 | 1 ± 0 | 1 ± 0 | 1 ± 0 | 0 ± 0 | 0 ± 0 | 0 ± 0 | 0 ± 0 | 1 ± 0 | 1 ± 0 |
|  |  | Cras1a | 9 ± 0 | 0 ± 0 | 40.5 ± 1.0 | 0 ± 0 | 1 ± 0 | 1 ± 0 | 1 ± 0 | 1 ± 0 | 0 ± 0 | 0 ± 0 | 0 ± 0 | 0 ± 0 | 1 ± 0 | 1 ± 0 |
|  |  | Cras1b | 1.5 ± 0.55 | 0 ± 0 | 48.0 ± 0.89 | 0 ± 0 | 1 ± 0 | 1 ± 0 | 1 ± 0 | 1 ± 0 | 0 ± 0 | 0 ± 0 | 0 ± 0 | 0 ± 0 | 1 ± 0 | 1 ± 0 |
|  |  | Cras2 | 2 ± 0 | 0 ± 0 | 47.5 ± 1.0 | 0 ± 0 | 1 ± 0 | 1 ± 0 | 1 ± 0 | 1 ± 0 | 0 ± 0 | 0 ± 0 | 0 ± 0 | 0 ± 0 | 1 ± 0 | 1 ± 0 |
|  |  | D | 8.5 ± 0.55 | 0 ± 0 | 41.0 ± 0.89 | 0 ± 0 | 1 ± 0 | 1 ± 0 | 1 ± 0 | 1 ± 0 | 0 ± 0 | 0 ± 0 | 0 ± 0 | 0 ± 0 | 1 ± 0 | 1 ± 0 |
|  |  | E | 4 ± 0 | 0 ± 0 | 45.5 ± 1.0 | 0 ± 0 | 1 ± 0 | 1 ± 0 | 1 ± 0 | 1 ± 0 | 0 ± 0 | 0 ± 0 | 0 ± 0 | 0 ± 0 | 1 ± 0 | 1 ± 0 |
|  |  | G | 2 ± 0 | 0 ± 0 | 47.5 ± 1.0 | 0 ± 0 | 1 ± 0 | 1 ± 0 | 1 ± 0 | 1 ± 0 | 0 ± 0 | 0 ± 0 | 0 ± 0 | 0 ± 0 | 1 ± 0 | 1 ± 0 |
|  |  | U | 2.5 ± 0.55 | 0 ± 0 | 47.0 ± 1.3 | 0 ± 0 | 1 ± 0 | 1 ± 0 | 1 ± 0 | 1 ± 0 | 0 ± 0 | 0 ± 0 | 0 ± 0 | 0 ± 0 | 1 ± 0 | 1 ± 0 |
|  | Test | A | 3 ± 0 | 0 ± 0 | 46.5 ± 1.0 | 0 ± 0 | 1 ± 0 | 1 ± 0 | 1 ± 0 | 1 ± 0 | 0 ± 0 | 0 ± 0 | 0 ± 0 | 0 ± 0 | 1 ± 0 | 1 ± 0 |
|  |  | B | 1.5 ± 0.55 | 0 ± 0 | 48.0 ± 0.89 | 0 ± 0 | 1 ± 0 | 1 ± 0 | 1 ± 0 | 1 ± 0 | 0 ± 0 | 0 ± 0 | 0 ± 0 | 0 ± 0 | 1 ± 0 | 1 ± 0 |
|  |  | C | 15.5 ± 0.55 | 0 ± 0 | 34.0 ± 0.89 | 0 ± 0 | 1 ± 0 | 1 ± 0 | 1 ± 0 | 1 ± 0 | 0 ± 0 | 0 ± 0 | 0 ± 0 | 0 ± 0 | 1 ± 0 | 1 ± 0 |
|  |  | Cras1a | 9 ± 0 | 0 ± 0 | 40.5 ± 1.0 | 0 ± 0 | 1 ± 0 | 1 ± 0 | 1 ± 0 | 1 ± 0 | 0 ± 0 | 0 ± 0 | 0 ± 0 | 0 ± 0 | 1 ± 0 | 1 ± 0 |
|  |  | Cras1b | 1.5 ± 0.55 | 0 ± 0 | 48.0 ± 0.89 | 0 ± 0 | 1 ± 0 | 1 ± 0 | 1 ± 0 | 1 ± 0 | 0 ± 0 | 0 ± 0 | 0 ± 0 | 0 ± 0 | 1 ± 0 | 1 ± 0 |
|  |  | Cras2 | 2 ± 0 | 0 ± 0 | 47.5 ± 1.0 | 0 ± 0 | 1 ± 0 | 1 ± 0 | 1 ± 0 | 1 ± 0 | 0 ± 0 | 0 ± 0 | 0 ± 0 | 0 ± 0 | 1 ± 0 | 1 ± 0 |
|  |  | D | 8.5 ± 0.55 | 0 ± 0 | 41.0 ± 0.89 | 0 ± 0 | 1 ± 0 | 1 ± 0 | 1 ± 0 | 1 ± 0 | 0 ± 0 | 0 ± 0 | 0 ± 0 | 0 ± 0 | 1 ± 0 | 1 ± 0 |
|  |  | E | 4 ± 0 | 0 ± 0 | 45.5 ± 1.0 | 0 ± 0 | 1 ± 0 | 1 ± 0 | 1 ± 0 | 1 ± 0 | 0 ± 0 | 0 ± 0 | 0 ± 0 | 0 ± 0 | 1 ± 0 | 1 ± 0 |
|  |  | G | 2 ± 0 | 0 ± 0 | 47.5 ± 1.0 | 0 ± 0 | 1 ± 0 | 1 ± 0 | 1 ± 0 | 1 ± 0 | 0 ± 0 | 0 ± 0 | 0 ± 0 | 0 ± 0 | 1 ± 0 | 1 ± 0 |
|  |  | U | 2.5 ± 0.55 | 0 ± 0 | 47.0 ± 1.3 | 0 ± 0 | 1 ± 0 | 1 ± 0 | 1 ± 0 | 1 ± 0 | 0 ± 0 | 0 ± 0 | 0 ± 0 | 0 ± 0 | 1 ± 0 | 1 ± 0 |
| PVY | Training | C | 8 ± 0 | 0 ± 0 | 117.0 ± 0.89 | 0 ± 0 | 1 ± 0 | 1 ± 0 | 1 ± 0 | 1 ± 0 | 0 ± 0 | 0 ± 0 | 0 ± 0 | 0 ± 0 | 1 ± 0 | 1 ± 0 |
|  |  | N | 15.5 ± 0.55 | 0 ± 0 | 109.5 ± 0.55 | 0 ± 0 | 1 ± 0 | 1 ± 0 | 1 ± 0 | 1 ± 0 | 0 ± 0 | 0 ± 0 | 0 ± 0 | 0 ± 0 | 1 ± 0 | 1 ± 0 |
|  |  | NTN | 36.5 ± 0.55 | 0 ± 0 | 88.5 ± 0.55 | 0 ± 0 | 1 ± 0 | 1 ± 0 | 1 ± 0 | 1 ± 0 | 0 ± 0 | 0 ± 0 | 0 ± 0 | 0 ± 0 | 1 ± 0 | 1 ± 0 |
|  |  | N:O | 18.5 ± 0.55 | 0 ± 0 | 106.5 ± 1.0 | 0 ± 0 | 1 ± 0 | 1 ± 0 | 1 ± 0 | 1 ± 0 | 0 ± 0 | 0 ± 0 | 0 ± 0 | 0 ± 0 | 1 ± 0 | 1 ± 0 |
|  |  | O | 43.5 ± 0.55 | 0 ± 0 | 81.5 ± 1.0 | 0 ± 0 | 1 ± 0 | 1 ± 0 | 1 ± 0 | 1 ± 0 | 0 ± 0 | 0 ± 0 | 0 ± 0 | 0 ± 0 | 1 ± 0 | 1 ± 0 |
|  |  | Poha | 3 ± 0 | 0 ± 0 | 122.0 ± 0.89 | 0 ± 0 | 1 ± 0 | 1 ± 0 | 1 ± 0 | 1 ± 0 | 0 ± 0 | 0 ± 0 | 0 ± 0 | 0 ± 0 | 1 ± 0 | 1 ± 0 |
|  | Test | C | 8 ± 0 | 0 ± 0 | 117.0 ± 0.89 | 0 ± 0 | 1 ± 0 | 1 ± 0 | 1 ± 0 | 1 ± 0 | 0 ± 0 | 0 ± 0 | 0 ± 0 | 0 ± 0 | 1 ± 0 | 1 ± 0 |
|  |  | N | 15.5 ± 0.55 | 0 ± 0 | 109.5 ± 0.55 | 0 ± 0 | 1 ± 0 | 1 ± 0 | 1 ± 0 | 1 ± 0 | 0 ± 0 | 0 ± 0 | 0 ± 0 | 0 ± 0 | 1 ± 0 | 1 ± 0 |
|  |  | NTN | 36.5 ± 0.55 | 0 ± 0 | 88.5 ± 0.55 | 0 ± 0 | 1 ± 0 | 1 ± 0 | 1 ± 0 | 1 ± 0 | 0 ± 0 | 0 ± 0 | 0 ± 0 | 0 ± 0 | 1 ± 0 | 1 ± 0 |
|  |  | N:O | 18.5 ± 0.55 | 0 ± 0 | 106.5 ± 1.0 | 0 ± 0 | 1 ± 0 | 1 ± 0 | 1 ± 0 | 1 ± 0 | 0 ± 0 | 0 ± 0 | 0 ± 0 | 0 ± 0 | 1 ± 0 | 1 ± 0 |
|  |  | O | 43.5 ± 0.55 | 0 ± 0 | 81.5 ± 1.0 | 0 ± 0 | 1 ± 0 | 1 ± 0 | 1 ± 0 | 1 ± 0 | 0 ± 0 | 0 ± 0 | 0 ± 0 | 0 ± 0 | 1 ± 0 | 1 ± 0 |
|  |  | Poha | 3 ± 0 | 0 ± 0 | 122.0 ± 0.89 | 0 ± 0 | 1 ± 0 | 1 ± 0 | 1 ± 0 | 1 ± 0 | 0 ± 0 | 0 ± 0 | 0 ± 0 | 0 ± 0 | 1 ± 0 | 1 ± 0 |
