# Supplementary material for: Clasnip: a web-based intraspecies classifier and multi-locus sequence typing for pathogenic microorganisms using fragmented sequences
Source: PeerJ. 2023 Jan 9;11:e14490. doi: 10.7717/peerj.14490 (PMC9835710; doi:10.7717/peerj.14490)
Supplement: Supplemental Information 4 [file peerj-11-14490-s004.docx]

**Table S4:**

**The classification result of Clasnip and BLCA of 16S rRNA sequences.**

| **Real Haplotype** | **Accession ID** | **BLCA Result** | **Clasnip Result** |
| --- | --- | --- | --- |
| A | EU834130.1 | A | A |
| A | JX624246.1 | A | A |
| A | KR935886.1 | A | A |
| A | KR935887.1 | A | A |
| A | MK726035.1 | A | A |
| A | MK726036.1 | A | A |
| B | JF811596.1 | B | B |
| B | JF811597.1 | B | B |
| B | KU588194.1 | B | B |
| B | KU588195.1 | B | B |
| B | MK726032.1 | B | B |
| B | MK726033.1 | B | B |
| B | MK726034.1 | B | B |
| B | MK726037.1 | B | B |
| C | KF170062.1 | C | C |
| C | KF170063.1 | C | C |
| C | KF170065.1 | C | C |
| C | KF170066.1 | C | C |
| C | KX431889.1 | Unclassified | C |
| C | KX431890.1 | Unclassified | C |
| C | KX431891.1 | Unclassified | C |
| C | KY624595.1 | C | C |
| C | MG701017.1 | C | U |
| Cras1a | MT229445.1 | Cras2 | Cras1a |
| Cras1a | MT229447.1 | Cras1a | Cras1a |
| Cras1a | MT229448.1 | Cras1a | Cras1a |
| Cras1a | MT229449.1 | Cras1a | Cras1a |
| Cras1a | MT229452.1 | Cras1a | Cras1a |
| Cras1a | MT229453.1 | Cras1a | Cras1a |
| Cras1a | MT229454.1 | Cras1a | Cras1a |
| Cras1a | MT229455.1 | Cras1a | Cras1a |
| Cras1a | MT229456.1 | Cras1a | Cras1a |
| Cras1a | MT229457.1 | Cras1a | Cras1a |
| Cras1a | MT229458.1 | Cras1a | Cras1a |
| Cras1a | MT229459.1 | Cras1a | Cras1a |
| Cras1a | MT229460.1 | Cras1a | Cras1a |
| Cras1b | MT229446.1 | Cras2 | Cras1b |
| Cras1b | MT229450.1 | Cras1a | Cras1b |
| Cras1b | MT229451.1 | Cras1a | Cras1b |
| Cras2 | MT229461.1 | Cras2 | Cras2 |
| Cras2 | MT229462.1 | Cras2 | Cras2 |
| Cras2 | MT229463.1 | Cras1a | Cras2 |
| D | KX163276.1 | D | D |
| D | KX163277.1 | D | D |
| D | KX752587.1 | D | D |
| D | KY486296.1 | C | D |
| D | KY624596.1 | D | D |
| D | MG701014.1 | D | D |
| D | MG911711.1 | C | D |
| D | MG911712.1 | C | D |
| D | MH061376.1 | C | D |
| E | KX752588.1 | E | E |
| E | KX752589.1 | E | E |
| E | KY619991.1 | E | E |
| E | KY619992.1 | E | E |
| E | MG911713.1 | E | E |
| F | MH259699.1 | F | F |
| G | MN256493.1 | G | G |
| G | MN256494.1 | G | G |
| G | MN256495.1 | G | G |
| H | MK800167.1 | H | H |
| H-Con | KT354971.1 | H-Con | H-Con |
| H-Con | KT354975.1 | H-Con | H-Con |
| U | MG701016.1 | C | U |
