# Supplementary material for: Clasnip: a web-based intraspecies classifier and multi-locus sequence typing for pathogenic microorganisms using fragmented sequences
Source: PeerJ. 2023 Jan 9;11:e14490. doi: 10.7717/peerj.14490 (PMC9835710; doi:10.7717/peerj.14490)
Supplement: Supplemental Information 5 — TPR = Sensitivity, Recall, Hit Rate, True Positive Rate. TNR = Specificity, Selectivity, True Negative Rate. PPV = Precision, Positive Predictive Value. NPV = Negative Predictive Value. FNR = Miss Rate, False Negative Rate. FPR = Fall-out, False Positive Rate. FDR = False Discovery Rate. FOR = False Omission Rate. ACC = Accuracy. F1 = the harmonic mean of precision and sensitivity. [file peerj-11-14490-s005.docx]

**Table S5:**

**Performance comparison of Clasnip and BLCA using CLso 16S rRNA sequences.**

TPR = Sensitivity, Recall, Hit Rate, True Positive Rate.
TNR = Specificity, Selectivity, True Negative Rate.
PPV = Precision, Positive Predictive Value.
NPV = Negative Predictive Value.
FNR = Miss Rate, False Negative Rate.
FPR = Fall-out, False Positive Rate.
FDR = False Discovery Rate.
FOR = False Omission Rate.
ACC = Accuracy.
F1 = the harmonic mean of precision and sensitivity.

| **Program** | **Haplotype** | **TPR** | **TNR** | **PPV** | **NPV** | **FNR** | **FPR** | **FDR** | **FOR** | **ACC** | **F1** |
| --- | --- | --- | --- | --- | --- | --- | --- | --- | --- | --- | --- |
| Clasnip | A | 1.000 | 1.000 | 1.000 | 1.000 | 0.000 | 0.000 | 0.000 | 0.000 | 1.000 | 1.000 |
|  | B | 1.000 | 1.000 | 1.000 | 1.000 | 0.000 | 0.000 | 0.000 | 0.000 | 1.000 | 1.000 |
|  | C | 0.889 | 1.000 | 1.000 | 0.982 | 0.111 | 0.000 | 0.000 | 0.018 | 0.984 | 0.941 |
|  | Cras1a | 1.000 | 1.000 | 1.000 | 1.000 | 0.000 | 0.000 | 0.000 | 0.000 | 1.000 | 1.000 |
|  | Cras1b | 1.000 | 1.000 | 1.000 | 1.000 | 0.000 | 0.000 | 0.000 | 0.000 | 1.000 | 1.000 |
|  | Cras2 | 1.000 | 1.000 | 1.000 | 1.000 | 0.000 | 0.000 | 0.000 | 0.000 | 1.000 | 1.000 |
|  | D | 1.000 | 1.000 | 1.000 | 1.000 | 0.000 | 0.000 | 0.000 | 0.000 | 1.000 | 1.000 |
|  | E | 1.000 | 1.000 | 1.000 | 1.000 | 0.000 | 0.000 | 0.000 | 0.000 | 1.000 | 1.000 |
|  | F | 1.000 | 1.000 | 1.000 | 1.000 | 0.000 | 0.000 | 0.000 | 0.000 | 1.000 | 1.000 |
|  | G | 1.000 | 1.000 | 1.000 | 1.000 | 0.000 | 0.000 | 0.000 | 0.000 | 1.000 | 1.000 |
|  | H | 1.000 | 1.000 | 1.000 | 1.000 | 0.000 | 0.000 | 0.000 | 0.000 | 1.000 | 1.000 |
|  | H-Con | 1.000 | 1.000 | 1.000 | 1.000 | 0.000 | 0.000 | 0.000 | 0.000 | 1.000 | 1.000 |
|  | U | 1.000 | 0.984 | 0.500 | 1.000 | 0.000 | 0.016 | 0.500 | 0.000 | 0.984 | 0.667 |
| BLCA | A | 1.000 | 1.000 | 1.000 | 1.000 | 0.000 | 0.000 | 0.000 | 0.000 | 1.000 | 1.000 |
|  | B | 1.000 | 1.000 | 1.000 | 1.000 | 0.000 | 0.000 | 0.000 | 0.000 | 1.000 | 1.000 |
|  | C | 0.667 | 0.909 | 0.545 | 0.943 | 0.333 | 0.091 | 0.455 | 0.057 | 0.875 | 0.600 |
|  | Cras1a | 0.923 | 0.941 | 0.800 | 0.980 | 0.077 | 0.059 | 0.200 | 0.020 | 0.938 | 0.857 |
|  | Cras1b | 0.000 | 1.000 | NaN | 0.953 | 1.000 | 0.000 | NaN | 0.047 | 0.953 | 0.000 |
|  | Cras2 | 0.667 | 0.967 | 0.500 | 0.983 | 0.333 | 0.033 | 0.500 | 0.017 | 0.953 | 0.571 |
|  | D | 0.556 | 1.000 | 1.000 | 0.932 | 0.444 | 0.000 | 0.000 | 0.068 | 0.938 | 0.714 |
|  | E | 1.000 | 1.000 | 1.000 | 1.000 | 0.000 | 0.000 | 0.000 | 0.000 | 1.000 | 1.000 |
|  | F | 1.000 | 1.000 | 1.000 | 1.000 | 0.000 | 0.000 | 0.000 | 0.000 | 1.000 | 1.000 |
|  | G | 1.000 | 1.000 | 1.000 | 1.000 | 0.000 | 0.000 | 0.000 | 0.000 | 1.000 | 1.000 |
|  | H | 1.000 | 1.000 | 1.000 | 1.000 | 0.000 | 0.000 | 0.000 | 0.000 | 1.000 | 1.000 |
|  | H-Con | 1.000 | 1.000 | 1.000 | 1.000 | 0.000 | 0.000 | 0.000 | 0.000 | 1.000 | 1.000 |
|  | U | 0.000 | 1.000 | NaN | 0.984 | 1.000 | 0.000 | NaN | 0.016 | 0.984 | 0.000 |
