# Supplementary material for: Clasnip: a web-based intraspecies classifier and multi-locus sequence typing for pathogenic microorganisms using fragmented sequences
Source: PeerJ. 2023 Jan 9;11:e14490. doi: 10.7717/peerj.14490 (PMC9835710; doi:10.7717/peerj.14490)
Supplement: Supplemental Information 6 [file peerj-11-14490-s006.docx]

**Table S6:**

**Clasnip job IDs of real CLso sample classification**

| **Sample** | **Real haplotype** | **Region** | **Clasnip Job ID** |
| --- | --- | --- | --- |
| J2 | A | 16S | fe215358-39ad-55db-80f7-38b23d14c824.clso_v5_genomic |
| J3 | A | 16S | 344357d5-b2a4-55df-9004-3a89a720400c.clso_v5_genomic |
| J5 | A | 16S | d816a7e2-abce-539b-bf23-07209662cc2b.clso_v5_genomic |
| J6 | A | 16S | d5f56166-e014-5f58-9324-6f34f44509ec.clso_v5_genomic |
| J8 | A | 16S | 1c578eae-53eb-58d6-9421-ab79a90938c1.clso_v5_genomic |
| J9 | A | 16S | 925df4ae-2170-5fa5-b408-98e8d2669e57.clso_v5_genomic |
| M1 | B | 16S + 50S | d302f514-b997-5be0-9e32-24c1dc9d4823.clso_v5_genomic |
| M2 | B | 16S + 50S | d16b2b88-f723-5956-a8ae-709b0533165c.clso_v5_genomic |
| M3 | B | 16S + 50S | 2bacb4c4-d2a1-58b4-a4d2-f171d0710663.clso_v5_genomic |
| M5 | B | 16S + 50S | 23dfb097-7ab0-5fc3-8012-72106cc9c6ed.clso_v5_genomic |
| M6 | B | 16S + 50S | dd4a7b23-0544-5c81-aa1f-ef139e3476dc.clso_v5_genomic |
| M7 | B | 16S + 50S | f90b0964-93ab-5bb2-88aa-ad478dab2446.clso_v5_genomic |
| M8 | B | 16S + 50S | 73e8ad62-3435-51db-bb69-cf08ac970c0e.clso_v5_genomic |
| M9 | A | 16S + 50S | 2b7deba4-b830-5bcf-a08d-735fd0ecfc5e.clso_v5_genomic |
| M10 | B | 16S + 50S | e06b9a1d-1d0e-543b-bf44-021483fafa76.clso_v5_genomic |
