# Supplementary material for: Clasnip: a web-based intraspecies classifier and multi-locus sequence typing for pathogenic microorganisms using fragmented sequences
Source: PeerJ. 2023 Jan 9;11:e14490. doi: 10.7717/peerj.14490 (PMC9835710; doi:10.7717/peerj.14490)
Supplement: Supplemental Information 7 [file peerj-11-14490-s007.docx]

**Table S7:**

**The accession numbers of PVY samples used in Clasnip database building**

| **PVY Phylogroup** | **NCBI Accession ID** |
| --- | --- |
| C | AF237963.1 |
| C | AF463399.1 |
| C | AJ439544.1 |
| C | AJ439545.1 |
| C | AJ890348.1 |
| C | DQ309028.1 |
| C | EU482153.1 |
| C | EU563512.1 |
| C | KF770835.1 |
| C | KP063211.1 |
| C | KP691320.1 |
| C | KP691321.1 |
| C | KP691324.1 |
| C | KP691329.1 |
| C | KR528584.1 |
| C | KX580384.1 |
| Chile3 | FJ214726.1 |
| N | AB331515.1 |
| N | AB331516.1 |
| N | AB331517.1 |
| N | AB331518.1 |
| N | AB331519.1 |
| N | AB711144.1 |
| N | AB714135.1 |
| N | AJ585197.1 |
| N | AJ585198.1 |
| N | AJ890346.1 |
| N | AM268435.1 |
| N | AY166866.1 |
| N | AY166867.1 |
| N | AY884983.1 |
| N | AY884984.1 |
| N | CS434575.1 |
| N | DQ157180.1 |
| N | HQ912867.1 |
| N | JN936422.1 |
| N | JQ969036.1 |
| N | JQ971975.1 |
| N | KR270797.1 |
| N | KT290511.1 |
| N | KT290512.1 |
| N | KU724101.1 |
| N | KX531041.1 |
| N | KY847984.1 |
| N | KY847985.1 |
| N | KY847986.1 |
| N | KY847988.1 |
| N | X97895.1 |
| N:O | AJ584851.1 |
| N:O | AJ889868.1 |
| N:O | AJ890349.1 |
| N:O | AJ890350.1 |
| N:O | AM113988.1 |
| N:O | AY745491.1 |
| N:O | AY745492.1 |
| N:O | AY884985.1 |
| N:O | DQ008213.1 |
| N:O | DQ157178.1 |
| N:O | DQ157179.1 |
| N:O | EF026076.1 |
| N:O | HE608963.1 |
| N:O | HE608964.1 |
| N:O | HM991454.1 |
| N:O | HQ912862.1 |
| N:O | HQ912863.1 |
| N:O | HQ912866.1 |
| N:O | HQ912868.1 |
| N:O | HQ912870.1 |
| N:O | HQ912871.1 |
| N:O | HQ912872.1 |
| N:O | HQ912896.1 |
| N:O | JF795485.1 |
| N:O | JF927750.1 |
| N:O | JF927751.1 |
| N:O | JF927753.1 |
| N:O | JF927754.1 |
| N:O | JF927755.1 |
| N:O | JF927758.1 |
| N:O | JF927760.1 |
| N:O | JF927762.1 |
| N:O | JQ924286.1 |
| N:O | JQ969039.1 |
| N:O | JQ969040.1 |
| N:O | JQ969041.1 |
| N:O | KJ634023.1 |
| NTN | AB185833.1 |
| NTN | AB270705.1 |
| NTN | AB461450.1 |
| NTN | AB461451.1 |
| NTN | AB461452.1 |
| NTN | AB461453.1 |
| NTN | AB461454.1 |
| NTN | AB702945.1 |
| NTN | AB711143.1 |
| NTN | AB711145.1 |
| NTN | AB711146.1 |
| NTN | AJ585342.1 |
| NTN | AJ889866.1 |
| NTN | AJ889867.1 |
| NTN | AJ890342.1 |
| NTN | AJ890343.1 |
| NTN | AJ890344.1 |
| NTN | AJ890345.1 |
| NTN | AJ890347.1 |
| NTN | AY884982.1 |
| NTN | EF016294.1 |
| NTN | EF026075.1 |
| NTN | FJ204164.1 |
| NTN | FJ204165.1 |
| NTN | FJ204166.1 |
| NTN | GQ200836.1 |
| NTN | HG810949.1 |
| NTN | HG810950.1 |
| NTN | HG810951.1 |
| NTN | HG810952.1 |
| NTN | HM590405.1 |
| NTN | HM590406.1 |
| NTN | HQ631374.1 |
| NTN | HQ912869.1 |
| NTN | JF927749.1 |
| NTN | JF927752.1 |
| NTN | JF927756.1 |
| NTN | JF927757.1 |
| NTN | JF927759.1 |
| NTN | JF927761.1 |
| NTN | JF927763.1 |
| NTN | JF928458.1 |
| NTN | JF928459.1 |
| NTN | JF928460.1 |
| NTN | JQ673517.1 |
| NTN | JQ924287.1 |
| NTN | JQ969033.1 |
| NTN | JQ969034.1 |
| NTN | JQ969035.1 |
| NTN | JQ969037.1 |
| NTN | KC296433.1 |
| NTN | KC296434.1 |
| NTN | KC296435.1 |
| NTN | KC296436.1 |
| NTN | KC296437.1 |
| NTN | KC296438.1 |
| NTN | KC296439.1 |
| NTN | KC296440.1 |
| NTN | KC296441.1 |
| NTN | KC614702.1 |
| NTN | KC634004.1 |
| NTN | KC634005.1 |
| NTN | KC634006.1 |
| NTN | KC634007.1 |
| NTN | KC634008.1 |
| NTN | KC634009.1 |
| NTN | KF850513.1 |
| NTN | KJ603224.1 |
| NTN | KJ603225.1 |
| NTN | KJ634024.1 |
| NTN | KJ946936.1 |
| NTN | KM396648.1 |
| NTN | M95491.1 |
| O | A08776.1 |
| O | AB711147.1 |
| O | AB711148.1 |
| O | AB711149.1 |
| O | AB711150.1 |
| O | AB711151.1 |
| O | AB711152.1 |
| O | AB711153.1 |
| O | AB711154.1 |
| O | AB711155.1 |
| O | AB714134.1 |
| O | AF522296.1 |
| O | AJ585195.1 |
| O | AJ585196.1 |
| O | CS434577.1 |
| O | D00441.1 |
| O | EF026074.1 |
| O | EF558545.1 |
| O | FJ643477.1 |
| O | FJ643478.1 |
| O | FJ643479.1 |
| O | HM367075.1 |
| O | HM367076.1 |
| O | HM590407.1 |
| O | HQ912864.1 |
| O | HQ912865.1 |
| O | HQ912873.1 |
| O | HQ912874.1 |
| O | HQ912875.1 |
| O | HQ912876.1 |
| O | HQ912877.1 |
| O | HQ912878.1 |
| O | HQ912879.1 |
| O | HQ912880.1 |
| O | HQ912881.1 |
| O | HQ912882.1 |
| O | HQ912883.1 |
| O | HQ912884.1 |
| O | HQ912885.1 |
| O | HQ912886.1 |
| O | HQ912887.1 |
| O | HQ912888.1 |
| O | HQ912889.1 |
| O | HQ912890.1 |
| O | HQ912891.1 |
| O | HQ912892.1 |
| O | HQ912893.1 |
| O | HQ912894.1 |
| O | HQ912895.1 |
| O | HQ912897.1 |
| O | HQ912898.1 |
| O | HQ912899.1 |
| O | HQ912900.1 |
| O | HQ912901.1 |
| O | HQ912902.1 |
| O | HQ912903.1 |
| O | HQ912904.1 |
| O | HQ912905.1 |
| O | HQ912906.1 |
| O | HQ912907.1 |
| O | HQ912908.1 |
| O | HQ912909.1 |
| O | HQ912910.1 |
| O | HQ912911.1 |
| O | HQ912912.1 |
| O | HQ912913.1 |
| O | HQ912914.1 |
| O | HQ912915.1 |
| O | JQ663997.1 |
| O | JQ924285.1 |
| O | JX424837.1 |
| O | KP691317.1 |
| O | KP691318.1 |
| O | KP691319.1 |
| O | KP691322.1 |
| O | KP691323.1 |
| O | KP691325.1 |
| O | KP691326.1 |
| O | KP691327.1 |
| O | KP691328.1 |
| O | KP691330.1 |
| O | KY847962.1 |
| O | KY848004.1 |
| O | KY848012.1 |
| O | NC_001616.1 |
| O | U09509.1 |
| O | X12456.1 |
| Poha | MF134861.1 |
| Poha | MF134862.1 |
| Poha | MF134863.1 |
| Poha | MF134864.1 |
| Poha | MF134865.1 |
| Poha | MF134866.1 |
